# Ethyl Oleate Synthesized from Fermentation Waste and Its Stability Evaluation as a Food Additive

**DOI:** 10.3390/foods15081382

**Published:** 2026-04-16

**Authors:** Ana Luiza Fontes, Ana Maria S. Soares, Francisca S. Teixeira, Paula T. Costa, Lígia L. Pimentel, Luís Miguel Rodríguez-Alcalá

**Affiliations:** Universidade Católica Portuguesa, CBQF—Centro de Biotecnologia e Química Fina—Laboratório Associado, Escola Superior de Biotecnologia, Rua Diogo Botelho 1327, 4169-005 Porto, Portugal; afontes@ucp.pt (A.L.F.); amsoares@ucp.pt (A.M.S.S.); fsteixeira@ucp.pt (F.S.T.); ptcosta@ucp.pt (P.T.C.)

**Keywords:** high oleic waste, ethyl oleate, waste valorisation, food additive, stability

## Abstract

Ethyl oleate (EO) is an emerging compound used in the food industry as a pre-treatment additive in preservation processes, such as drying, allowing the shelf-life to be extended while preserving the nutritional value of the treated food without compromising consumer safety. Currently, EO is mostly synthesised from edible oils, which raises concerns about competition with the food chain. As an alternative, we previously developed an EO product from a *High-Oleic Waste* (*HOW*) obtained from industrial distillation pipelines. Due to the potential application of EO as a food additive, the present study aimed to evaluate its stability throughout its shelf-life in comparison with two commercial benchmarks under accelerated conditions (40 °C, 75% relative humidity, 6 months). Colour parameters (*Total Colour Difference* and *Yellow Index*), structural properties by FTIR-ATR, thermal properties by DSC, compositional stability by GC-MS, formation of lipid oxidation products by UV-Vis and cytotoxicity in keratinocytes were evaluated at the beginning (T0) and at the end (T6) of the assay. In general, the synthesised EO showed no considerable changes in the parameters studied after storage, being comparable to the assayed benchmarks. In conclusion, the developed EO was found to be stable during the assayed shelf-life, confirming its potential suitability as an additive for the food industry. Future studies should perform validation in food matrices.

## 1. Introduction

Currently, sustainable practices are essential for the conservation of available resources. In this context, food loss and waste generation have become a major concern for society. Extending the shelf-life of food products represents an essential strategy, promoting, in the meantime, better food quality, greater food safety and enhanced consumer protection [1]. Thus, the development of more efficient preservation processes became a major goal of the food industry worldwide [2]. Among the main methods applied nowadays (e.g., freezing, smoking, vacuum, salting, modified atmosphere, or high-pressure conservation), drying is one of the oldest and most widely used [3]. This process consists of reducing the moisture content to a level that allows safe storage of a product for a longer period [4,5,6]. The removal of water from foods is essential to minimise or inhibit microbial growth and deterioration reactions, as well as to help maintain the nutritional value of food. There are several drying methodologies, including freeze, hot-air, heat pump, cabinet and tray drying, among others, that can be applied either in isolation or in combination [5]. These conservation processes offer additional benefits, namely, reduced packaging requirements and shipping costs, achieved through the decreased weight and volume of the dried product [7]. However, despite the wide range of drying methods available, depending on the characteristics of the food product, the process may require long operational times, reducing its sustainability in terms of energy consumption. To overcome this downside, preservatives are usually applied prior to the drying procedure. Beyond their protective role, these compounds also contribute to texture and appearance preservation, flavour enhancement and nutrient fortification, without compromising food quality or safety [8]. An emerging additive that combines such functional and technological properties is ethyl oleate (EO). It is currently used in the pre-treatment of small fruits (e.g., blueberries, plums, mulberries) with a waxy peel structure that hinders the drying process [4,9,10,11,12]. In addition to shortening the drying time of this type of fruit, EO is also an important flavouring agent [13], with documented antioxidant and antimicrobial properties [4,14]. EO is a fatty acid ethyl ester, typically synthesised from high-oleic feedstocks, namely, vegetable oils [15], through two chemical processes. The first involves direct esterification of fatty acids with ethanol catalysed by acid [16,17,18,19]. The second one exploits the high triglyceride content of oils through transesterification with ethanol in the presence of an alkaline catalyst, such as sodium hydroxide (NaOH) [20,21,22]. In both processes, heating, an excess of ethanol and a catalyst are required to increase reaction kinetics and yield [21,22,23]. However, the use of vegetable oils as a raw material to produce EO raises concerns regarding competition with food availability. Therefore, recently, in the context of the circular economy, there has been increasing interest in developing products from renewable sources and industrial waste streams to reduce ecological footprints and CO_2_ emissions [24,25]. In that sense, previous work by our research team identified an underexplored waste effluent, termed *High-Oleic Waste (HOW)*, as a raw material for EO production [26]. Through optimised transesterification conditions, an EO product of high yield and purity was obtained from *HOW*, offering a sustainable alternative that avoids competition with the food chain. The environmental impact of the synthesis process, including solvent and reagent requirements, was evaluated. It was established that the utilisation of food-grade solvents, high recovery rates of reagents (approximately 97%), and implementation of an ethyl oleate recirculation strategy collectively support an environmentally sustainable production process aligned with circular economy principles. That work already identified the need for subsequent stability evaluation as a prerequisite for confirming the suitability of the product for food applications. Indeed, for any novel ingredient to be considered suitable as a food additive, its stability throughout its shelf-life must be demonstrated. This is a critical requirement, as the functional and safety properties of the ingredient must remain consistent from production to consumption. Despite the use of EO as a food additive, there is a significant lack of scientific data regarding its long-term stability under conditions relevant to food applications. Published work on EO degradation has been conducted either at elevated temperatures under controlled oxygen partial pressure to determine autoxidation kinetic parameters [27], using oxidative stress tests developed for biodiesel quality assessment [28], or through thermogravimetric analysis under inert atmospheres, aimed at characterising thermal degradation for commercial biodiesel applications [29]. These studies were conducted using high-purity chemical standards and were not designed to evaluate the stability of commercial products or those synthesised from a complex industrial waste stream.

Thus, the present study addresses this research gap and aims to assess the stability of the previously developed EO during storage under accelerated conditions, using commercial benchmarks as references.

## 2. Materials and Methods

### 2.1. Chemicals and Samples

The *High-Oleic Waste* (*HOW*) was kindly provided by Amyris, Inc. (Emeryville, CA, USA). EO Croda and EO Croda SR, used in this study as benchmark references, were kindly donated by Croda International Plc (DN149AA, East Yorkshire, United Kingdom). *N,O*-Bis(trimethylsilyl)trifluoroacetamide with 1% trimethylchlorosilane (BSTFA) was purchased from Merck (Darmstadt, Germany). Dichloromethane (DCM, HPLC-grade, ≥99.9%), ethanol (EtOH, food-grade, 96%), ethyl acetate (EtAc, food-grade, 99%), citric acid (C_6_H_8_O_7_, analytical-grade, 99%), sodium chloride (NaCl, analytical-grade, 99%) and sodium hydroxide pellets (NaOH, analytical-grade, 98%) were purchased from VWR Chemicals (Radnor, PA, USA). Cyclohexane (analytical-grade, ≥99.8%) was obtained from Carlo Erba Reagents (Val de Reuil, France).

### 2.2. Ethyl Oleate Synthesis

According to conditions previously optimised [26], the synthesis of EO was carried out with 9 g of *HOW* and 1 g of previously synthesised EO (*HOW*:EO—9:1, *w*/*w*) in a round-bottom flask, to which ethanol was added (EtOH:*HOW* ratio—6:1, *w*/*w*) and 1% (*w*/*w*) of ethanolic solution of NaOH (2.5 M). The mixture was heated to 70 °C under stirring for 3 h. Afterwards, the ethanol was evaporated by rotary evaporation (Heidolph HeiVAP; Schwalbach, Germany) and collected for reuse (~97% recovery, *v*/*v*). The crude mixture was neutralised with 1% (*w*/*v*) citric acid aqueous solution, and the resulting fatty acid ethyl esters (FAEEs) were extracted in a separation funnel with ethyl acetate (3 × 30 mL) and 3% (*w*/*v*) NaCl aqueous solution (30 mL). The organic phase was evaporated in a rotary evaporator, and the ethyl acetate was recovered (~98%, *v*/*v*). The FAEE mixture was dried in a ThermoFisher Scientific Oven (Waltham, MA, USA) overnight at 50 °C to remove any trace amounts of solvents.

### 2.3. Accelerated Storage Stability Assessment

The World Food Programme establishes that storing food samples at 40 °C and 75% RH for 6 months, with an assumed Q10 of 2, is equivalent to approximately 24 months of shelf-life at 20 °C ± 2 °C [30]. These conditions are further supported by the European Medicines Agency (EMA) guidelines on the stability testing of new drug substances and products [31], which independently define 40 °C ± 2 °C/75% RH ± 5% RH for 6 months as the standard accelerated condition for stability evaluation, providing additional regulatory basis for the experimental design adopted here.

Accordingly, approximately 3 mL of the synthesised EO (EO61R) and benchmarks (EO Croda and EO Croda SR) were stored in closed glass amber vials, in duplicate, under the above-mentioned conditions. At the beginning (T0) and the end (T6) of the study, aliquots of each sample were collected and stored at −80 °C until further analysis.

### 2.4. Colour Parameters

Colour coordinates (L*, a* and b*) were evaluated using a Minolta CR-410 colourimeter (Konica-Minolta, Osaka, Japan). The colorimeter was properly calibrated with a standard white calibration plate. Perceptual lightness was measured by the parameter L*, ranging from black (0) to white (100). Colour parameters a* (from green [−128] to red [+127]) and b* (from blue [−128] to yellow [+127]) were also recorded [32]. The *Total Colour Difference* (*TCD*) was determined as described by de Araújo et al. [33] and Ordóñez-Santos et al. [34]:

(1) *TCD* = (ΔL^2^ + Δa^2^ + Δb^2^)^1/2^

Moreover, the *Yellow Index* (*YI*), or Yellowness, was determined as described by Albisu et al. [35]:

(2) *YI* = (142.86 × b)/L

All analytical measurements were performed in triplicate as technical replicates for each sample.

### 2.5. Fourier Transform Infrared Spectroscopy–Attenuated Total Reflectance (FTIR-ATR) Spectra

Fourier Transform Infrared Spectroscopy–Attenuated Total Reflectance (FTIR-ATR) spectra were acquired on a PerkinElmer Paragon 1000 FTIR (Waltham, MA, USA) with an ATR accessory, incorporated with a Diamond/ZnSe crystal. The FTIR-ATR measurements were performed in transmittance mode within the wavenumber range of 4000–550 cm^−1^, with a 4 cm^−1^ resolution by the accumulation of 16 scans. Data processing was performed using the software Spectrum 10.1.0. All analytical measurements were performed in triplicate as technical replicates for each sample.

### 2.6. Differential Scanning Calorimetry (DSC) Measurements

Differential Scanning Calorimetry (DSC) measurements were performed using a NE-TZSCH DSC 204 F1 Phoenix (NETZSCH-Gerätebau GmbH, Selb, Germany) calorimeter. Samples (2–3 mg) were placed into aluminium crucibles that were subsequently sealed. Runs were performed as a cycle of various alternating heating and cooling steps to determine the melting, crystallisation, and degradation temperatures of the samples, as follows: step 1—heating from 20 °C to 110 °C, step 2—cooling from 110 °C to −60 °C, step 3—heating from −60 °C to 250 °C, step 4—cooling from 250 °C to −60 °C and step 5—heating from −60 °C to 500 °C. Each step was performed at a rate of 10 °C/min, including an isothermal step of 1 min at the end of each heating/cooling step. A nitrogen flow rate of 100 mL/min was maintained during the runs, and an empty and sealed crucible was used as a control. The indicated data were extracted from steps 3 to 5; steps 1 and 2 were performed to eliminate possible moisture content. All analytical measurements were performed in triplicate as technical replicates for each sample.

### 2.7. Lipid Oxidation Product Evaluation by UV Spectroscopy

To evaluate the possible formation of products resulting from lipid oxidation, the International Olive Council (COI/T.20/Doc. No 19/Rev. 5 2019) has approved a methodology based on determining the specific extinction coefficient (K) by UV spectroscopy according to Grau et al. [36] and Velasco et al. [37]. As such, each sample was dissolved in cyclohexane at 1% (*w*/*v*), and UV spectra were acquired at the fixed wavelengths of 270 nm and 232 nm. Then, when needed, successive dilutions and respective measurements were performed until the absorbance values were between 0.1 and 0.8. The determination of specific extinctions at 270 nm (K_270_) and 232 nm (K_232_) was calculated as follows:

(3) K_λ_ = E_λ_/c × s,

where K_λ_ is the specific extinction coefficient at wavelength λ; E_λ_ is the absorbance measured at wavelength λ; c is the concentration of the solution in g/100 mL; and s is the path length of the quartz cell in cm.

The determination of the specific extinction variation (ΔK) is given below:

(4) ΔK = K_m_ − [(K_m−4_ + K_m+4_)/2],

where K_m_ is the specific extinction at the wavelength for maximum absorption at 232 nm and 270 nm. ΔK values, whether positive or negative, reflect deviations from spectral ideality rather than the magnitude of absorption at the specific wavelength (232 or 270 nm), and therefore should not be interpreted as a direct measure of compound concentration. All analytical measurements were performed in triplicate as technical replicates for each sample.

### 2.8. Gas Chromatography–Mass Spectrometry (GC-MS) Analysis

Samples were first derivatised into their trimethylsilyl derivatives. Thus, 1 mg of a sample was accurately weighed and added to 500 µL of DCM and 30 µL of BSTFA. After 60 min of incubation at 30 °C, DCM was added to a final volume of 1.5 mL. The derivatised samples were analysed on a GC-MS model EVOQ (Bruker, Karlsruhe, Germany) coupled to a mass spectrometer, with a Rxi-5Sil MS column (Restek Corporation, Bellefonte, PA, USA) (30 m × 250 µm × 0.25 µm) at a constant flow of 1 mL/min. The carrier gas used was helium (He), and the GC-MS conditions were as described by Teixeira et al. [38] with slight modifications. The injector was set to 340 °C with a 1:10 split, and the oven temperature started at 60 °C with a hold for 1 min, increasing at a rate of 5 °C/min until 340 °C and maintained for 10 min. The MS detector was operated in electron ionisation mode (EI) at 70 eV, with a source temperature of 280 °C, the transfer line at 300 °C, and a quadrupole in a scan range from 33 to 1000 amu per second. The sample composition was assessed by relative abundance. All analytical measurements were performed in triplicate as technical replicates for each sample.

### 2.9. High-Performance Liquid Chromatography (HPLC) Coupled to an Evaporative Light Scattering Detector

For a broader lipid analysis, the Plante et al. (2011) [39] methodology was used with some modifications. Approximately 3 mg/mL of a sample dissolved in a methanol:chloroform mixture (1:1, *v*/*v*) was injected in a high-performance liquid chromatograph (HPLC) (1260 Infinity II; Agilent, Santa Clara, CA, USA) equipped with an evaporative light scattering detector (ELSD) and a Zorbax Eclipse Plus C8 column (2.1 × 100 mm; Agilent). The detector operation conditions were set with the evaporator at 30 °C, the nebuliser at 40 °C, and nitrogen as carrier gas with a flow rate set at 1.5 SLM. The mobile phases were prepared and filtered by a 0.20 µm pore size hydrophobic membrane: A (methanol, water and acetic acid, 750:250:4 (*v*/*v*)) and B (acetonitrile, methanol, tetrahydrofuran, and acetic acid, 500:375:125:4 (*v*/*v*)) in gradient mode were set between 0 and 45 min (100% Phase A, 46–59 min—30% Phase A and 70% Phase B, 60–65 min—10% Phase A and 90% Phase B, and 65.10–72 min—100% Phase A). The determination was carried out by injecting 10 µL of the sample at a flow rate of 0.5 mL/min with the oven set to 40 °C. All analytical measurements were performed in triplicate as technical replicates for each sample.

### 2.10. Cytotoxicity Assessment

During handling and application of EO as a food additive, dermal contact is likely to occur. Therefore, cytotoxicity was assessed using a human keratinocyte cell line as an indicator of skin safety under handling conditions. The human keratinocyte cell line HaCaT (CLS—Cell Line Services—300493) was evaluated using the PrestoBlue assay according to the manufacturer’s instructions (Thermofisher, Waltham, MA, USA). The cell line was kept in culture in Dulbecco’s Modified Eagle Medium (DMEM; Thermofisher, Waltham, MA, USA) supplemented with 10% Fetal Bovine Serum (FBS) (Thermofisher, Waltham, MA, USA) and 1% penicillin–streptomycin antibiotic (Thermofisher, Waltham, MA, USA) at 37 °C, with 5% CO_2_ in a humidified atmosphere. The cells were seeded at 1 × 10^4^ cells/well in 96-well plates and exposed to the EO samples at different concentrations (0.1 to 5 mg/mL diluted in DMEM) for 24 h, in quadruplicate (i.e., four replicate wells per concentration). Two independent experiments were performed (n = 2).

Additionally, to confirm the possible cytotoxicity of EO61R (i.e., at T0 and T6), macrophages were evaluated using a PrestoBlue assay (Thermofisher, Waltham, MA, USA), according to the manufacturer’s instructions. THP-1 cells (ATCC TIB-202) were seeded at 1 × 10^4^ cells/well in 96-well plates and differentiated into macrophages by treatment with 50 nM phorbol 12-myristate 13-acetate (PMA) for 48 h, as described by Teixeira et al. [40]. Then, the cells were exposed to EO61R at different concentrations (0.1 to 5 mg/mL diluted in DMEM) for 24 h, in quadruplicates (i.e., four replicate wells per concentration). Two independent experiments were performed (n = 2).

In both experiments, wells with media supplemented with lipidic extracts (without cells) were used to subtract the possible influence of the samples on the PrestoBlue fluorescence signal. Cells treated with 10% Dimethyl Sulfoxide (DMSO) (Thermofisher, Waltham, MA, USA) were used as a negative control. After incubation, PrestoBlue was added to the media and incubated for 2 h. The fluorescence signal was read using a Synergy H1 microplate reader (BioTek, Winooski, VT, USA). Results were expressed as metabolic inhibition percentage compared to the control (cells without treatment). Values above 30% were considered cytotoxic.

### 2.11. Statistical Analysis

Results are reported as mean values ± standard deviations. Data were first analysed for normality (i.e., Shapiro–Wilk). Levene’s test was applied to verify the homogeneity of variances. Afterwards, a one-way ANOVA test was applied with a Bonferroni post hoc test to compare groups. To determine differences from T0 to T6, we performed the T-student test for paired samples. The level of significance was generally set at 0.05. Analyses were performed with the aid of IBM SPSS Statistics software (28.0 version, Chicago, IL, USA).

## 3. Results and Discussion

### 3.1. Colour Parameter Stability by CIELAB Colorimetry

#### 3.1.1. Total Colour Difference (TCD)

The colour coordinates recorded for each EO sample are presented in Table 1, as well as the calculated *TCD*.

The evaluation of *TCD* gives information about how different the sample colour is before (T0) and after (T6) storage. If the value of *TCD* is ˂1.5, a small colour difference is detected; if 1.5 < *TCD* < 3, the change in colour is considered distinct; and if *TCD* is >3, it means that the colour is very distinct [41]. According to this scale, for all ethyl oleates tested, a small difference in their colours could be observed between T0 and T6 (0.46 ± 0.03 for EO61R, 0.43 ± 0.11 for EO Croda and 0.83 ± 0.01 for EO Croda SR). Compared with the benchmarks, the colour difference of EO61R after storage was similar to that of EO Croda, but lower than that of EO Croda SR (*p* < 0.05).

#### 3.1.2. *Yellow Index (YI)*

The *YI* describes the change in colour of a tested sample from clear or white to yellow. Since the EO samples were yellow in colour, the determination of this parameter became relevant. The obtained results are presented in Table 2. A significant increase in *YI* was observed in the commercial samples (EO Croda and EO Croda SR), with a variation of 1.00 ± 0.02 and 2.38 ± 0.12 units, respectively. In contrast, for EO61R, a slight decrease (*p* < 0.05) was observed. The negative ΔYI observed for EO61R reflects a modest reduction in yellow intensity from an already elevated baseline value (YI = 25.45 ± 0.40 at T0), which is markedly higher than that of the benchmark samples (0.60 ± 0.01 and 0.31 ± 0.02, respectively). This difference in baseline YI is attributable to the complex composition of EO61R, which includes minor components such as partial glycerides and free oleic acid that are absent in the commercial benchmarks (Tables 5 and 6).

The YI increases observed in the benchmark samples suggest a shift in colour within the yellow range during storage. While oxidative processes are frequently cited as a cause of colour deepening in lipid-based systems, through the formation of conjugated carbonyl structures and oxidised acylglycerol derivatives [42], the lipid oxidation data (Section 3.4) and compositional analyses (Section 3.5) do not support significant oxidative deterioration in any of the samples under the storage conditions assayed. It should be noted that colour changes in refined lipid-based products can also arise from the interaction of minor nonacylglycerol components, such as tocopherol oxidation derivatives, with oxidised triacylglycerols, a phenomenon documented in refined vegetable oils independent of primary lipid oxidation indicators [43]. A full mechanistic characterisation of the colour changes observed, including the role of specific minor components, would require kinetic studies and structural analyses such as NMR, which are beyond the scope of the present work and represent a relevant direction for future investigation.

### 3.2. Structural Stability by FTIR-ATR

**Figure 1:**
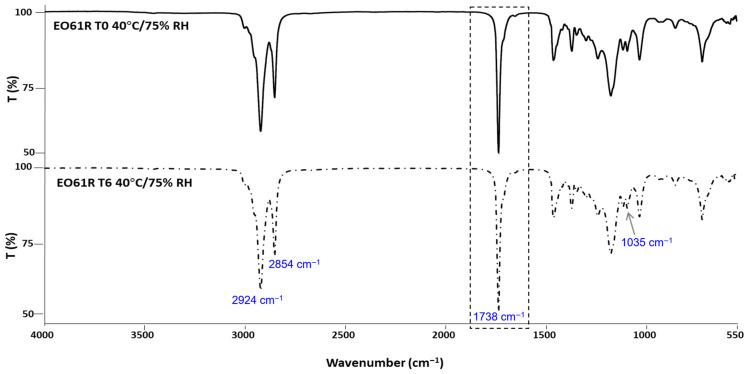
FTIR-ATR spectra of EO61R before (T0) and after 6 months (T6) of storage under accelerated conditions (40 °C/75% RH).

**Figure 2:**
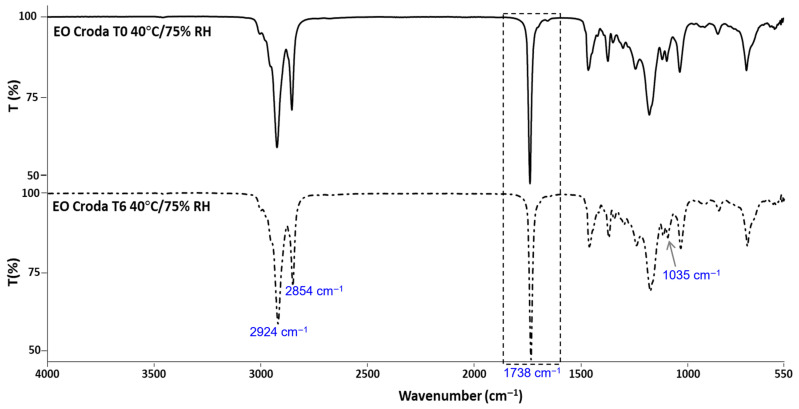
FTIR-ATR spectra of EO Croda before (T0) and after 6 months (T6) of storage under accelerated conditions (40 °C/75% RH).

**Figure 3:**
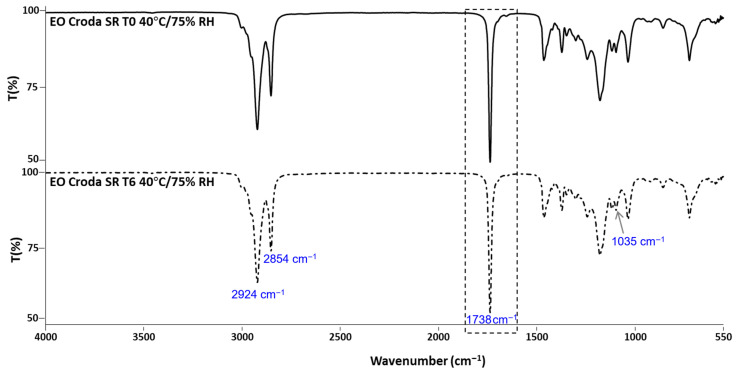
FTIR-ATR spectra of EO Croda SR before (T0) and after 6 months (T6) of storage under accelerated conditions (40 °C/75% RH).

The results obtained by FTIR analysis of the EO61R (Figure 1), EO Croda (Figure 2) and EO Croda SR samples (Figure 3) are shown in the corresponding figures. The main absorption bands are consistent with those previously reported for EO61R at the time of synthesis [26]. Accordingly, the most characteristic band, assigned to the C=O stretching vibration of fatty acid ethyl esters, was observed at 1738 cm^−1^ [44], in agreement with values reported by Soares et al. [26] (1737 cm^−1^) and Niu et al. [29] (1738 cm^−1^) for oleic acid ethyl ester. Additional characteristic bands include those at 2924 and 2854 cm^−1^, corresponding to the C–H axial deformation of the olefinic double bond and the anti-symmetric and symmetric stretching vibrations of CH_2_ and CH_3_ groups, respectively [26,29], and the band at approximately 1035 cm^−1^, attributable to the C–O–C symmetric stretching vibration specific to the ethyl ester group [29].

No changes in band position or intensity were detected between T0 and T6 for any of the three samples. The carbonyl band at ~1738 cm^−1^ remained unaffected throughout storage in all cases, indicating that the ester bond was not cleaved or chemically modified under the conditions assayed. Additionally, no bands characteristic of oxidation-related structural changes were detected. Daoud et al. [45] reported that oxidation of unsaturated lipids in fatty acid-based systems is accompanied by a progressive attenuation of the bands at ~3012 and ~2927 cm^−1^ and by an increase at ~968 cm^−1^, associated with the C–H out-of-plane deformation of trans double bonds, reflecting cis-to-trans isomerisation of unsaturated acyl chains. Furthermore, the absence of any detectable band in the 3400 cm^−1^ region, where OH stretching vibrations of hydroperoxides would be expected [45], further indicates that primary oxidation products did not accumulate to measurable levels during storage. The absence of these spectral changes in the present study is consistent with the low oxidation indicators determined by UV spectroscopy (Section 3.4) and the absence of compositional changes observed by GC-MS (Section 3.5).

### 3.3. Thermal Stability by DSC

The thermal transition results of all EO samples during storage are presented in Table 3. Two events were observed for EO61R: at −44.0 ± 0.2 °C (T0) and −4.2 ± 0.3 °C (T0) and at −49.1 ± 0.3 °C and −4.4 ± 0.2 °C at T6. One melting event was detected at −23.4 ± 0.2 °C (T0) and −24.1 ± 0.2 °C (T6), and degradation occurred at 295.0 ± 5.5 °C (T0) and at 297.7 ± 2.1 °C (T6).

The presence of two crystallisation events in EO61R, which are absent in the commercial benchmarks, can be explained by its compositional profile, as further presented in the GC-MS and HPLC-ELSD results (Tables 5 and 6). The primary crystallisation event at approximately −44 °C is consistent with the bulk ethyl oleate fraction, whose crystallisation temperature has been reported in the range −43 to −50 °C [26]. The second crystallisation event at approximately −4 °C can be attributed to minor components with distinct thermal properties.

This interpretation is consistent with previous studies on FAEE systems, which demonstrate that low-temperature behaviour is strongly governed by the highest-melting components present in the mixture [46]. In particular, saturated fatty acid ethyl esters exhibit significantly higher crystallisation temperatures and tend to dominate the onset of solid formation in multicomponent systems. GC-MS analysis of EO61R identified ethyl palmitate and ethyl stearate (please refer to Section 3.5), both saturated FAEEs with substantially higher melting points than ethyl oleate, alongside free oleic acid. These species are absent or present only at trace levels in the benchmark samples and are therefore consistent with the emergence of a higher-temperature crystallisation event.

More broadly, the formation of multiple crystallisation events is characteristic of FAEE mixtures exhibiting non-ideal solid–liquid equilibrium behaviour, including eutectic transitions and phase separation effects [47]. Such behaviour reflects the coexistence of components with markedly different melting points and crystallisation kinetics. In addition, HPLC-ELSD analysis detected monoglycerides and diglycerides in EO61R. These partial glycerides retain free hydroxyl groups that promote intermolecular hydrogen bonding, further modifying crystallisation pathways and contributing to the observed thermal complexity.

The combined presence of saturated FAEEs and partial glycerides therefore provides a compositionally consistent explanation for the additional crystallisation event observed exclusively in EO61R.

Comparison of T0 and T6 reveals a shift in the primary crystallisation temperature of EO61R (from −44.0 ± 0.2 °C to −49.1 ± 0.3 °C; *p* > 0.05), while the secondary crystallisation event, melting temperature, and degradation temperature remain unchanged. As GC-MS and HPLC-ELSD analyses showed no significant variation in the relative abundance of any detected component between T0 and T6 (Tables 5 and 6), this shift is unlikely to reflect chemical degradation of the bulk ethyl oleate fraction. Instead, the shift is more plausibly attributed to changes in crystallisation kinetics and nucleation behaviour within the multicomponent matrix. Previous work has shown that crystallisation in FAEE systems is highly sensitive to nucleation phenomena, where the initial formation of crystals can influence subsequent crystallisation pathways and temperature profiles [47]. A subtle redistribution or reorganisation of minor components during storage may therefore alter nucleation efficiency, leading to a measurable shift in the apparent crystallisation temperature without any detectable compositional change.

This interpretation is further supported by the absence of any corresponding change in the melting temperature, which is an equilibrium property and less sensitive to kinetic and matrix effects. No significant changes in any thermal parameter were observed for either benchmark between T0 and T6 (*p* > 0.05), consistent with their simpler and more homogeneous compositions.

A full mechanistic understanding of the crystallisation shift in EO61R, including the individual contributions of specific minor components, would require isothermal crystallisation studies and enthalpy-resolved modelling across multiple heating rates. Such approaches have been shown to be effective in describing FAEE phase behaviour and represent a relevant direction for future work.

### 3.4. Lipid Oxidation Products

Lipid oxidation is a major determinant of food quality and shelf-life, particularly in products rich in unsaturated fatty acids. Its consequences include flavour and aroma deterioration, nutritional value loss, notably of polyunsaturated fatty acids, and the potential generation of harmful compounds [48,49,50]. Given that EO is a fatty acid ethyl ester containing unsaturated bonds, it is susceptible to oxidation. The extent of oxidation can be quantified by UV–visible spectroscopy through the determination of specific extinction coefficients (K): K_232_ reflects the accumulation of primary oxidation products (peroxides, hydroperoxides, and conjugated dienes), while K_270_ is associated with secondary oxidation products (alcohols, ketones, aldehydes, and conjugated trienes) [51]. The values of specific extinction coefficients (K) and related variations (ΔK) obtained between T0 and T6 are presented in Table 4.

Regarding primary oxidation products (K_232_), a significant increase was observed in both benchmark samples after storage (*p* < 0.05), with the largest change recorded for EO Croda SR (from 2.32 ± 0.19 to 4.33 ± 0.28). In contrast, K_232_ values for EO61R remained stable between T0 and T6 (2.04 ± 0.01 and 2.25 ± 0.16, respectively). The variation parameter ΔK_232_ increased significantly in all samples (*p* < 0.05), but the magnitude was notably lower for EO61R (from 0.12 ± 0.01 to 0.28 ± <0.01) compared to the benchmarks.

With respect to secondary oxidation products (K_270_), values for EO61R were 0.63 ± 0.02 at T0 and 0.56 ± 0.05 at T6, while those for EO Croda decreased significantly from 1.79 ± 0.01 to 1.07 ± 0.04 (*p* < 0.05). For EO Croda SR, K_270_ remained low and essentially unchanged (0.19 ± 0.02 at T0 and 0.23 ± 0.02 at T6). Similarly, ΔK_270_ showed no significant variation across samples. The increase in K_232_ together with stable or slightly decreasing K_270_ values is consistent with early-stage oxidation, where primary oxidation products (conjugated dienes) are formed without significant generation of secondary oxidation compounds absorbing at 270 nm. However, it also has to be considered that the FTIR analyses have not detected bands related to oxidation.

The results obtained are consistent with previously reported data on fatty acid ester oxidative stability. Storage of fatty acid ethyl and methyl esters (FAEEs and FAMEs) derived from degummed sunflower oil for up to 90 days showed that the peroxide value, acid value, anisidine value, and UV absorbance at 232 nm all increased over time, in proportion to storage temperature (50 °C > 30 °C > 20 °C), with FAEEs oxidising more rapidly than FAMEs [52]. In a further study using FAMEs from refined high-oleic sunflower oil, K_232_ increased from 2.19 to 2.83 after 25 h at 40 °C, reaching 4.45 after 48 h, at which point the peroxide value exceeded 79 meq/kg [53]. In that study, samples were placed in open Petri dishes in an oven, with continuous exposure to air and light. By contrast, the EO samples in the present study were stored in closed amber vials, which limits both oxygen availability and light exposure, two primary drivers of lipid oxidation, and likely accounts for the lower K_232_ values observed here.

As a reference framework, EU regulations for olive oil establish maximum K_232_, K_270_, and ΔK values of <2.50, ≤0.22, and ≤0.01, respectively, for extra virgin olive oil, while refined olive oil is subject to limits only for K_270_ (≤1.10) and ΔK (≤0.20), with no defined threshold for K_232_ [54]. Although these limits apply to olive oil and cannot be directly extrapolated to ethyl oleate, they provide a useful framework for contextualising the oxidative status of lipid-based products. The K_232_ values recorded for EO61R at T0 and T6 were below the extra virgin olive oil threshold, whereas both benchmark samples exceeded it at T6, further supporting the oxidative stability of the synthesised EO under the storage conditions tested.

### 3.5. Compositional Profile Stability

The composition of the tested EO samples is presented in Table 5. Ethyl palmitate, linoleate, oleate and stearate, as well as cis-vaccenic and oleic acids, were detected in EO61R. The composition of the benchmarks was similar, but oleic acid was missing, while ethyl laurate was further detected in EO Croda SR. Ethyl oleate was the main component of EO61R (86.93 ± 0.09% at T0), followed by ethyl linoleate (3.36 ± 0.04% at T0) and oleic acid (3.23 ± 0.01% at T0). The percentage of ethyl oleate in EO Croda and EO Croda SR was lower (78.26 ± 0.06% and 78.08 ± 0.16% at T0, respectively) and contained more ethyl palmitate (9.64 ± 0.04% and 9.21 ± 0.07% at T0, respectively) and linoleate (8.70 ± 0.05% and 8.29 ± 0.06% at T0, respectively). Upon comparing T0 with T6, no significant (*p* > 0.05) differences were found in the ethyl oleate content or any of the other compounds detected in all the assayed samples.

The compositional stability of all samples was further confirmed by HPLC-ELSD analysis (Table 6). Consistent with the GC-MS results, no significant differences (*p* > 0.05) were observed in the relative abundance of any detected compound between T0 and T6 across all samples, with ethyl oleate remaining the predominant component throughout storage. Notably, the minor partial glyceride components detected in EO61R, namely, monoglycerides and diglycerides, remained unchanged between T0 and T6. These compounds, being partial esters retaining free hydroxyl groups and unsaturated acyl chains, would be particularly susceptible to further reaction or oxidative degradation. Their stability therefore provides additional support for the conclusion that no significant extensive chemical changes took place under the assayed conditions, confirming compositional stability independently of the analytical approach used.

The relative abundance of ethyl oleate determined by HPLC-ELSD was higher than that obtained by GC-MS for all samples, which is attributable to the different detection principles and compound coverage of the two techniques.

### 3.6. Metabolic Inhibition Evaluation on Keratinocytes

Given the intended application of the developed EO as a food additive, confirming the absence of cytotoxicity represents an important prerequisite before any food application study can be conducted. Thus, the cytotoxicity of EO61R and the EO benchmarks was assessed by monitoring changes in the degree of cell metabolic inhibition throughout storage.

**Figure 4:**
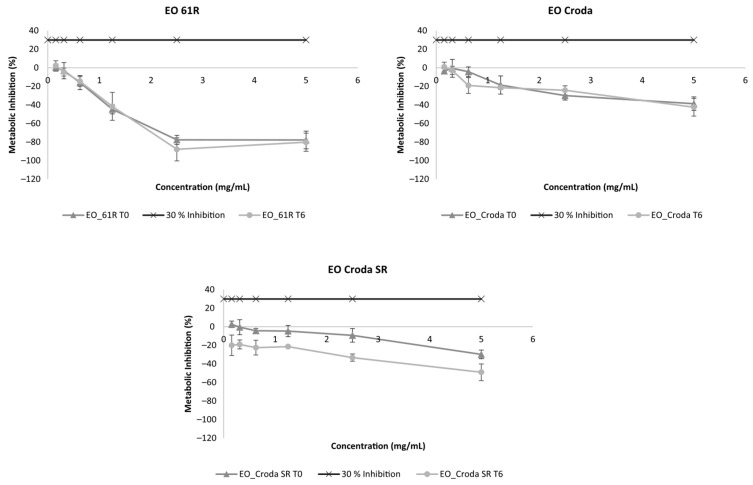
Metabolic inhibition (%) on HaCaT cells of ethyl oleate samples before (T0) and after 6 months (T6) of storage under accelerated conditions (40 °C/75% RH). Results are expressed as mean ± SD (n = 2).

The results regarding the metabolic inhibition of EO samples on keratinocytes (HaCaT) are presented in Figure 4. Metabolic inhibition of any of the EO samples at T0 was below 30% for all tested concentrations (up to 5 mg/mL). Furthermore, after storage, all EO samples showed no considerable differences in metabolic inhibition compared to T0.

**Figure 5:**
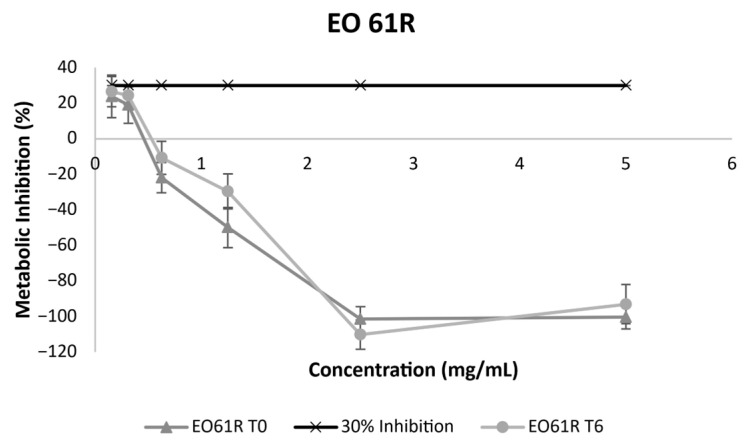
Metabolic inhibition (%) on THP-1-derived macrophages of EO61R before (T0) and after 6 months (T6) of storage under accelerated conditions. Results are expressed as mean ± SD (n = 2).

The cytotoxicity of EO61R was further assessed in a macrophage model (THP-1-derived) at T0 and T6 (Figure 5), providing complementary evidence to the HaCaT keratinocyte data. Metabolic inhibition remained below the 30% cytotoxicity threshold at all concentrations in both cell models and at both time points, with no significant differences observed between T0 and T6.

To the best of our knowledge, no previous published studies have evaluated the biocompatibility of EO using in vitro cell line models, making the present data a novel contribution to the safety characterisation of this compound. The available evidence on EO safety derives primarily from animal and human studies. A 91-day subchronic feeding study in Sprague Dawley rats at dietary concentrations up to 10% EO (approximately 6 g/kg/day) reported no toxicologically significant findings across a comprehensive panel of parameters including clinical observations, haematology, clinical chemistry, organ weights, and histopathology, with a NOAEL (i.e., No Observable Adverse Effect Level) established at the highest dose tested [55]. These findings were supported by a human clinical trial in which consumption of up to 16 g EO per day for 12 weeks produced no clinically significant adverse effects on any measured physiological or biochemical parameter [56]. Notably, Patel et al. [57] demonstrated that fatty acid ethyl esters, including EO (OAEE), were substantially less toxic than their parent free fatty acids at equimolar concentrations in pancreatic acinar cells and peripheral blood mononuclear cells in vitro and caused significantly milder pancreatitis than the corresponding triglyceride in an animal model.

The consistent absence of cytotoxicity observed in both HaCaT keratinocytes and THP-1-derived macrophages at T0 and T6 confirms that any minor chemical changes occurring under the assayed storage conditions did not result in the generation of compounds with cytotoxic activity. These in vitro results are consistent with the lack of toxicity reported in subchronic animal studies and human clinical trials, where EO was well tolerated at doses substantially higher than those anticipated in food additive applications [55,56,57]. The agreement between the present in vitro biocompatibility data and the previously reported in vivo evidence provides additional support for the safety profile of EO61R and its potential suitability as a food additive ingredient.

## 4. Conclusions

The physicochemical and biological stability of a previously developed ethyl oleate (EO61R) was assessed under accelerated storage conditions and compared with two commercial benchmarks.

With respect to colour, all samples exhibited minimal changes after storage (TCD < 1.5). However, the Yellow Index of EO61R decreased slightly, indicating a reduction in yellow intensity, whereas both benchmark samples showed the opposite trend, becoming more yellow over time.

Structural analysis by FTIR-ATR revealed no changes in the main absorption bands characteristic of fatty acid ethyl esters in any of the evaluated samples, confirming structural integrity throughout storage.

With respect to thermal stability, no significant changes were observed in the melting or degradation temperatures of EO61R. A significant shift was detected in one of the two temperatures of the crystallisation event, likely attributable to the presence of minor FAEE components in the sample; however, this did not reflect broader thermal instability.

Assessment of lipid oxidation products showed that the specific extinction coefficients K_232_ and K_270_ of EO61R remained largely unchanged after storage, indicating that neither primary nor secondary oxidation products were generated in significant quantities. In contrast, both benchmark samples exhibited increased oxidation indicators, particularly at 232 nm. The lipid composition of EO61R, as determined by GC-MS, was similarly unaffected by storage, with no significant differences observed in the relative abundance of any detected compound.

Cytotoxicity assessment in keratinocytes (HaCaT cells) confirmed that EO61R was non-cytotoxic at all tested concentrations, and this profile was maintained after storage, in line with the benchmark samples.

In summary, the assayed EO61R demonstrated stability across all evaluated parameters during accelerated shelf-life, comparable to the commercial ethyl oleates used in this study as reference benchmarks. These findings support the physicochemical and biological stability of EO61R under relevant storage, representing a necessary prerequisite for its future evaluation as a food additive in food application studies. Future research should focus on validating the performance and sensory impact of EO within specific food matrices, as well as conducting a comprehensive review of its compliance with regional regulatory frameworks (e.g., EFSA, FDA) prior to industrial application.

## Figures and Tables

**Table 1:** Colour coordinates (L*, a* and b*) and *Total Colour Difference* (*TCD*) of ethyl oleate samples before (T0) and after 6 months (T6) of storage under accelerated conditions.

Sample	Time	L* ^1^	a* ^1^	b* ^1^	*TCD* ^1,2^
EO61R	T0	42.24 ± 0.17	−0.15 ± 0.04	7.53 ± 0.15	0.46 ± 0.03 ^b^
	T6	42.40 ± 0.05	−0.26 ± 0.03	7.21 ± <0.01	
EO Croda	T0	44.24 ± 0.07	−0.29 ± 0.07	0.19 ± <0.01	0.43 ± 0.11 ^b^
	T6	44.48 ± 0.13	−0.34 ± 0.01	0.50 ± <0.01	
EO Croda SR	T0	44.54 ± 0.04	−0.19 ± 0.02	0.10 ± <0.01	0.83 ± 0.01 ^a^
	T6	44.39 ± 0.15	−0.47 ± 0.01	0.84 ± 0.04	

^1^ Average value ± standard deviation; n = 2. ^2^ Different superscript letters indicate significant differences (*p* < 0.05) between samples.

**Table 2:** *Yellow index* (*YI*) of ethyl oleate samples before (T0) and after 6 months (T6) of storage under accelerated conditions and observed variation (Δ*YI* = *YI*_T6_ − *YI*_T0_).

Sample	Time	*YI* ^1^	Δ*YI* ^1,2^
EO61R	T0	25.45 ± 0.40	−1.17 ± 0.32 ^c^
	T6	24.28 ± 0.03	
EO Croda	T0	0.60 ± 0.01	1.00 ± 0.02 ^b^
	T6	1.60 ± 0.01 *	
EO Croda SR	T0	0.31 ± 0.02	2.38 ± 0.12 ^a^
	T6	2.69 ± 0.11 *	

^1^ Average value ± standard deviation; n = 2. ^2^ Different superscript letters indicate significant differences (*p* < 0.05) between samples. * Significant differences among storage times within a sample.

**Table 3:** Temperature transitions obtained by DSC of ethyl oleate samples before (T0) and after 6 months (T6) of storage under accelerated conditions.

Sample	Time	Crystallisation (°C) ^1,2^			Melting (°C) ^1^			Degradation (°C) ^1^
EO61R	T0	−44.0 ± 0.2 ^a^	−4.2 ± 0.3			−23.4 ± 0.2		295.0 ± 3.9
	T6	−49.1 ± 0.3 ^b^	−4.4 ± 0.2			−24.1 ± 0.2		297.7 ± 1.5
EO Croda	T0	−46.6 ± 0.4		−15.4 ± 0.1	−25.8 ± 0.8	−22.3 ± 0.1	−7.1 ± <0.1	307.4 ± 2.9
	T6	−46.8 ± 0.1		−15.8 ± 0.1	−25.6 ± 0.1	−22.3 ± <0.1	−6.5 ± <0.1	293.6 ± 0.3
EO Croda SR	T0	−47.3 ± 0.1		−15.8 ± <0.1		−22.6 ± 0.3	−6.9 ± 0.3	308.7 ± 2.5
	T6	−47.4 ± 0.4		−15.8 ± 0.2		−22.6 ± <0.1	−5.7 ± 0.3	299.7 ± 1.5

^1^ Average value ± standard deviation; n = 2. ^2^ Different superscript letters indicate significant differences (*p* < 0.05) between T0 and T6.

**Table 4:** Specific extinction coefficients (K) at 232 nm and 270 nm and respective variations (ΔK_232_ and ΔK_270_) in ethyl oleate samples before (T0) and after 6 months (T6) of storage under accelerated conditions.

Sample	Time	K_232_ ^1,2^	ΔK_232_ ^1,2^	K_270_ ^1,2^	ΔK_270_ ^1,2^
EO61R	T0	2.04 ± 0.01	0.12 ± 0.01 ^b^	0.63 ± 0.02	0.05 ± <0.01
	T6	2.25 ± 0.16	0.28 ± <0.01 ^a^	0.56 ± 0.05	0.08 ± <0.01
EO Croda	T0	3.11 ± 0.04 ^b^	0.22 ± <0.01 ^b^	1.79 ± 0.01 ^a^	0.33 ± <0.01
	T6	3.93 ± 0.10 ^a^	0.78 ± <0.01 ^a^	1.07 ± 0.04 ^b^	0.33 ± <0.01
EO Croda SR	T0	2.32 ± 0.19 ^b^	0.28 ± 0.05 ^b^*	0.19 ± 0.02	−0.02 ± <0.01 ^b^*
	T6	4.33 ± 0.28 ^a^	0.83 ± 0.02 ^a^*	0.23 ± 0.02	−0.09 ± 0.02 ^a^*

^1^ Average value ± standard deviation; n = 2. ^2^ Different superscript letters indicate significant (*p* < 0.05) or tendential (* *p* < 0.10) differences between T0 and T6.

**Table 5:** Lipid composition (relative abundance, %) by GC-MS of ethyl oleate samples before (T0) and after 6 months (T6) of storage under accelerated conditions.

Compound	EO61R ^1^		EO Croda ^1^		EO Croda SR ^1^	
	T0	T6	T0	T6	T0	T6
Ethyl laurate	n.d. ^2^	n.d. ^2^	<LOQ ^2^	<LOQ ^2^	0.85 ± 0.02	0.86 ± 0.04
Ethyl palmitate	2.55 ± 0.02	2.54 ± 0.01	9.64 ± 0.04	9.51 ± 0.04	9.21 ± 0.07	9.26 ± 0.05
Ethyl linoleate	3.36 ± 0.04	3.33 ± 0.03	8.70 ± 0.05	8.10 ± 0.09	8.29 ± 0.06	8.06 ± 0.05
Ethyl oleate	86.93 ± 0.09	86.41 ± 0.14	78.26 ± 0.06	78.94 ± 0.15	78.08 ± 0.16	78.31 ± 0.16
cis-Vaccenic acid	1.38 ± 0.03	1.36 ± 0.03	1.84 ± 0.03	1.94 ± 0.03	2.04 ± 0.03	2.03 ± 0.04
Ethyl stearate	2.55 ± 0.01	2.52 ± 0.04	1.56 ± 0.01	1.52 ± 0.01	1.54 ± 0.04	1.49 ± 0.01
Oleic acid	3.23 ± 0.01	3.84 ± 0.08	n.d. ^2^	n.d. ^2^	n.d. ^2^	n.d. ^2^

^1^ Average value ± standard deviation; n = 2. ^2^ n.d.—not detected; <LOQ—below limit of quantification.

**Table 6:** Lipid composition (relative abundance, %) by HPLC-ELSD of ethyl oleate samples before (T0) and after 6 months (T6) of storage under accelerated conditions.

Compound	EO61R ^1^		EO Croda ^1^		EO Croda SR ^1^	
	T0	T6	T0	T6	T0	T6
Monoglyceride	2.40 ± 0.13	2.57 ± 0.07	n.d. ^2^	n.d. ^2^	n.d. ^2^	n.d. ^2^
Ethyl linoleate	1.85 ± 0.11	1.89 ± 0.05	7.25 ± 0.08	6.93 ± 0.08	6.87 ± 0.04	6.81 ± 0.08
Free fatty acid	0.23 ± 0.02	0.24 ± 0.02	2.77 ± 0.05	2.73 ± 0.02	2.43 ± 0.01	2.68 ± 0.07
Ethyl oleate	93.83 ± 0.36	93.57 ± 0.17	89.25 ± 0.09	89.63 ± 0.10	90.07 ± 0.02	89.84 ± 0.14
Ethyl stearate	1.27 ± 0.05	1.27 ± 0.02	0.73 ± 0.03	0.70 ± 0.01	0.63 ± 0.02	0.66 ± 0.01
Ethyl behenate	0.38 ± 0.04	0.39 ± 0.01	n.d. ^2^	n.d. ^2^	n.d. ^2^	n.d. ^2^
Diglyceride	0.06 ± 0.01	0.08 ± 0.01	n.d. ^2^	n.d. ^2^	n.d. ^2^	n.d. ^2^

^1^ Average value ± standard deviation; n = 2. ^2^ n.d.—not detected.

## Data Availability

The original contributions presented in this study are included in this article. Further inquiries can be directed to the corresponding authors.

## Abbreviations

The following abbreviations are used in this manuscript:

DCM	Dichloromethane
EO	Ethyl Oleate
FAEE	Fatty Acid Ethyl Ester
*HOW*	*High-Oleic Waste*
*TCD*	Total Colour Difference
*YI*	Yellow Index
